# Cancer Trends in Mexico: Essential Data for the Creation and Follow-Up of Public Policies

**DOI:** 10.1200/JGO.2016.007476

**Published:** 2017-03-15

**Authors:** Alejandro Mohar-Betancourt, Nancy Reynoso-Noverón, Daniel Armas-Texta, Cristina Gutiérrez-Delgado, Juan A. Torres-Domínguez

**Affiliations:** **Alejandro Mohar-Betancourt, Nancy Reynoso-Noverón, Daniel Armas-Texta, and Juan A. Torres-Domínguez,** National Cancer Institute; and **Cristina Gutiérrez-Delgado,** Ministry of Health, Cuidad de Mexico, Mexico.

## Abstract

**Purpose:**

Cancer in a country like Mexico is a challenge for the current health system and for public health. However, the statistics about cancer in Mexico are scarce, so epidemiologic surveillance needs to be improved. The objectives of this article were to describe the extent of cancer and to estimate the national burden of cancer through 2020.

**Materials and Methods:**

To meet this objective, an analysis of secondary official sources was performed. The cancer cases through 2020 were estimated on the basis of trends in mortality and the projection of incident cases reported by GLOBOCAN.

**Results:**

In 2013, cancer was the cause of 12.84% of all deaths in Mexico. It is projected that the prevalence of cancer will be 904,581 by 2017 and will reach 1,262,861 by early in the next decade (ie, 2020).

**Conclusion:**

Available data for cancer are incomplete. The development and implementation of population-based cancer registries in Mexico are essential. Assessment of the future outlook of cancer in Mexico will provide awareness of future challenges and can help health systems prepare to face them.

## INTRODUCTION

Cancer currently is one of the largest challenges in public health; it affects both developed and developing countries alike. However, in developing countries, the potential impact of cancer in future years may have devastating consequences for health systems.

Mexico, an upper-middle-income country, currently is going through an epidemiologic transition, in which mortality patterns are influenced by technological and medical developments. Currently, the main public health problems in the country are chronic degenerative diseases. Nevertheless, people in the country continue to experience significant morbidity and mortality as a result of nutritional deficiencies and infectious and contagious diseases.^1-4^

Epidemiologic surveillance in Mexico began under the Ministry of Health in the 1930s.^5^ Progress on communicable disease surveillance has been substantial, and the surveillance successfully addressed the threat of epidemic processes.^6,7^ However, surveillance of noncommunicable diseases—namely, cancer—is insufficient. The last attempt to record cancer occurrences was the Histopathological Registry of Malignant Neoplasms (RHNM).^8^ This registry ceased to function in 2003. Mexico does not have a population-based cancer registry; thus, data about the incidence and prevalence of the disease are lacking. Although reporting of new occurrences of different types of cancer has been promoted,^9^ fragmentation of the Mexican health system makes the full reporting of these cases inefficient; this inefficiency is the reason that only partial data about the extent of cancer in the country are available.^10^ The lack of data, in turn, hinders the creation and follow-up of public policies for the control of cancer.

The Central and South America (CSA) region is expected to experience an increase in the incidence of cancer to 1.7 million and a doubled mortality rate (500,000 to 1 million) by 2030. Likewise, the region will suffer a double burden of the disease: those cancers related to infections, and those cancers related to lifestyles.^11^

In this context, the current article uses national population projections and available cancer statistics to illustrate the burden entailed for Mexico in the forthcoming years. Similarly, opportunities to improve the collection of high-quality cancer data are discussed to emphasize the primary needs and the planning required to prepare for the upcoming cancer epidemic.

## MATERIALS AND METHODS

An analysis of secondary sources of cancer mortality and morbidity was performed. Official national sources of information were used. The mortality analysis included the International Classification of Disease (ICD) –10 codes of malignant neoplasms (C00 to C97, or corresponding codes in the ICD-9), carcinoma in situ (D00 to D09) and benign neoplasms and neoplasms of uncertain or unknown behavior (D10 to D48) for 2000 to 2013. These data were obtained from the Mexican National Institute of Statistics and Geography, available online in the National Health Information System.^12^ National mortality data were obtained from death certificates. Registered deaths were captured across the country in the Civil Registry System. These data are published annually, and the results have been presented at state, municipality, and locality levels since 1893. Morbidity estimates published by the GLOBOCAN system of the International Agency for Research on Cancer^13^ for the years 2002, 2008, and 2012 were collected.

### Mortality Analysis

Specific mortality rates by state and age group for the year 2013 are presented. In addition, the mortality trends by age and sex for the top 10 malignant diseases reported during the study period are presented.

Population projections from 2010 to 2050, published by the National Population Council, which consider births, deaths, and migration according to census data (2000 and 2010) and population and housing counts (1995 and 2005), were used.^14^ With the available data, estimates of the cancer mortality rate by age group and sex for the year 2020 were calculated in a conservative scenario. Trends were modeled by using linear regression analysis with curve fitting or smoothing with low-degree polynomials (ie, numerical polynomial spline interpolation methods).^15,16^ If the model violated or did not comply with any assumption, the forecasting was conducted through time series models.^17^

### Estimation of Prevalent Cases Until 2020

To estimate the prevalence of cancer, incident cases (published by GLOBOCAN) and deaths by sex and age group (0 to 14, 15 to 39, 40 to 44, 45 to 49, 50 to 54, 55 to 59, 60 to 64, 65 to 69, 70 to74, or ≥ 75 years) were considered with the following equation: *_x_*CEP*_t_^j^* = *_x_*CEI*_t_^j^* − *_x_*Dc*_t_^j^* + CEP_(_*_t_*_−1)_*^j^* in which CEP*_t_* = expected prevalent cancer cases at time *t*; CEI*_t_* = expected incident cancer cases at time *t*; Dc_t_ = deaths by cancer at time *t*; *x* = age group (*x* ∑ {1; 2;…; 10}); and *j* = Sex (*j* = 1, men; *j* = 2, women).

We assumed that the interval between one GLOBOCAN estimation and another would not change through the period with no data (eg, the incidence estimated for 2002 was used for 2002 to 2007; that estimated for 2008 was used for 2008 to 2011; and that estimated for 2012 was used for 2012 to 2020). It also was assumed that the data exhibited a logarithmic behavior for the incidence rate over time and the curve was adjusted with the ordinary least squares (OLS) method. Because there are no national incidence data, and because the latest national information available derives from the 2002 RHNM,^8^ we assumed that 50% of the new occurrences reported from the last figures from the histopathological registry (in 2002) remained alive at the time of our estimation; this number represented the initial prevalent cases.

Point values and CIs for each of the estimates are presented.^16,17^ The analysis was performed with R statistical software, version 3.1.

## RESULTS

### Cancer Mortality

In Mexico, cancer has remained the second or third leading cause of death. In 2000, cancer was responsible for 12.7% of deaths; in 2013, cancer accounted for 12.8% of all deaths and was the third leading cause of death in the country, after only heart disease (24.3%) and diabetes (14.3%). On average, the proportion was higher for women, and 45.4% of all cancer deaths occurred in the working-age population (15 to 64 years). When the usual place of residence is considered, noncommunicable diseases, such as cancer, have a higher mortality rate in northern Mexico.

**Fig 1:**
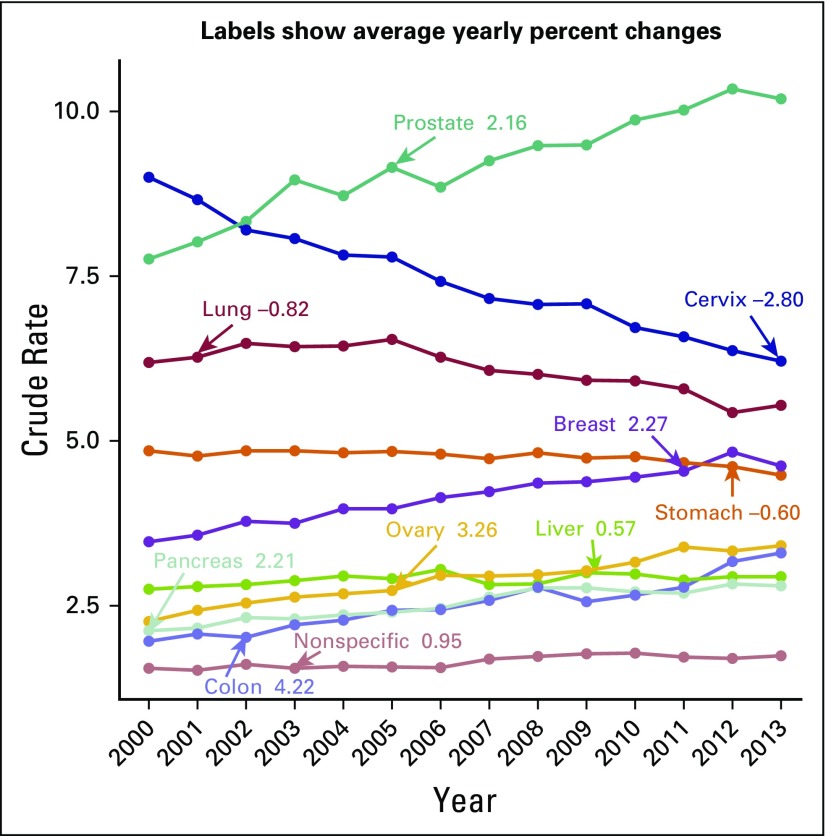
Mortality trends by cancer type per 100,000 people in Mexico, 2000-2013. Data used with permission.^12^

Cancer types with the highest absolute number of deaths between 2000 and 2013 were as follows: lung cancer (n = 6,678 average annual deaths), gastric cancer (n = 5,339), liver cancer (n = 4,931), prostate cancer (n = 4,859), breast cancer (n = 4,496), and cervical cancer (n = 4,181). Deaths from these six types of cancers accounted for 45% of all cancer-related deaths. The cancer mortality crude rate (per 100,000) rose from 58.7 in 2000 to 65.1 in 2013. The cancer mortality crude rate increased in men from 57.1 to 65.6,and in women from 57.1 to 64.7. The mortality rate trends and average percent changes during analyzed years by cancer type are shown in Figure 1. Specific mortality crude rates by sex for the 10 leading causes of cancer death between 2000 and 2013 are listed in Tables 1 and 2. Specific crude rates by age group for the 10 main malignant neoplasms in the years 2000, 2006, and 2013 show that the most affected age groups are those older than 40 years. A decrease in the mortality from cervical, lung, and stomach cancers was observed.

**Table 1:**
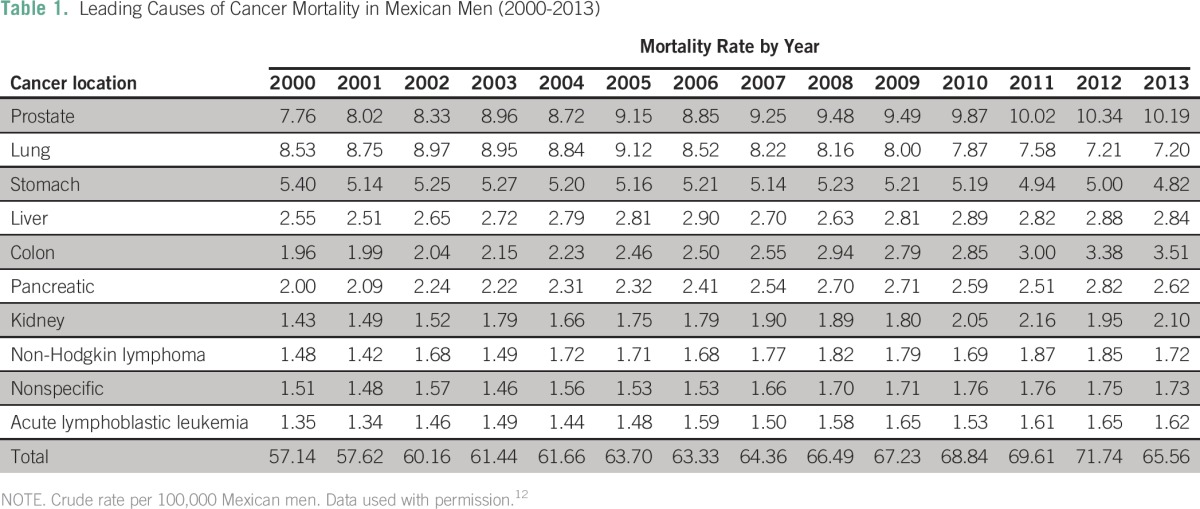
Leading Causes of Cancer Mortality in Mexican Men (2000-2013)

**Table 2:**
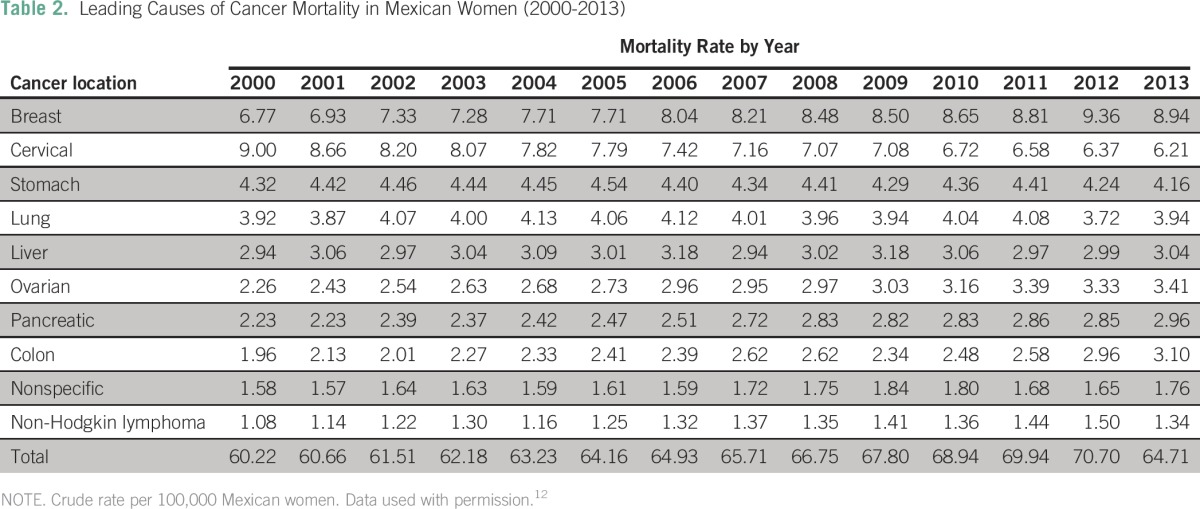
Leading Causes of Cancer Mortality in Mexican Women (2000-2013)

**Fig 2:**
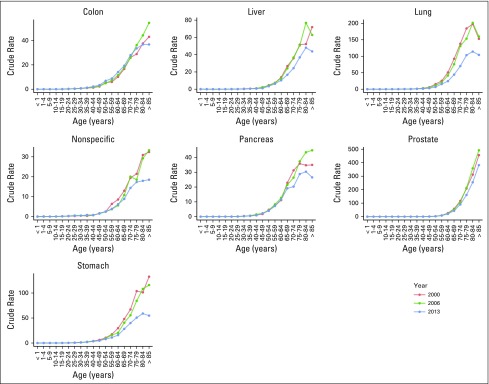
Age-specific cancer mortality (crude rate per 100,000) in Mexican men. Data used with permission.^12^

**Fig 3:**
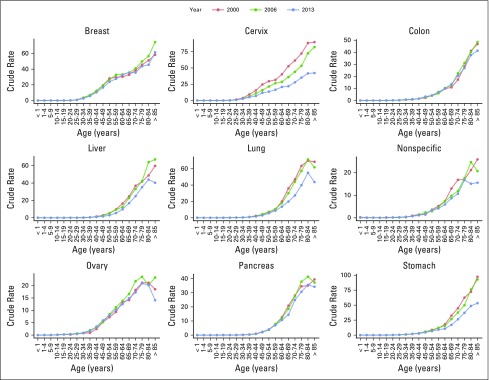
Age-specific cancer mortality (crude rate per 100,000) in Mexican women. Data used with permission.^12^

In men, prostate, lung, and gastric cancers had the highest mortality rates; crude rates increased for prostate cancer and remained stable for lung and gastric cancers (Fig 2). In women, breast, cervical, and gastric cancers had the highest cancer mortality crude rates, and this reflected a change from 2006 in the primary cause of death for women (from cervical to breast cancer; Fig 3).

In the population younger than age 20 years, the leading causes of death from neoplastic disease were acute lymphoblastic leukemia, acute myeloid leukemia, unspecified malignant brain tumor, cartilage tumors of bone and joints, and unspecified leukemia. These data have been previously described elsewhere.^18^

### Cancer Morbidity

Mexico does not track morbidity data. Official sources only provide information on the number of hospital discharges from public institutions without identification of patient cases. The only tumors for which incidence data are available are breast and cervical cancers; the crude incidence rates in 2012 were 16.99 and 8.59, respectively, per 100,000 women age 15 years or older. This rate is much lower than the estimated crude incidences of 34.7 and 32.9 per 100,000 women age 15 years and older for breast cancer and 23.7 and 10.5 per 100,000 women age 15 years and older for cervical cancer reported by GLOBOCAN and Sierra et al,^19^ respectively. The latest report of the 2002 RHNM included 108,545 new occurrences of cancer.^8^

### Estimation of the Extent of Cancer in Mexico by 2020

**Fig 4:**
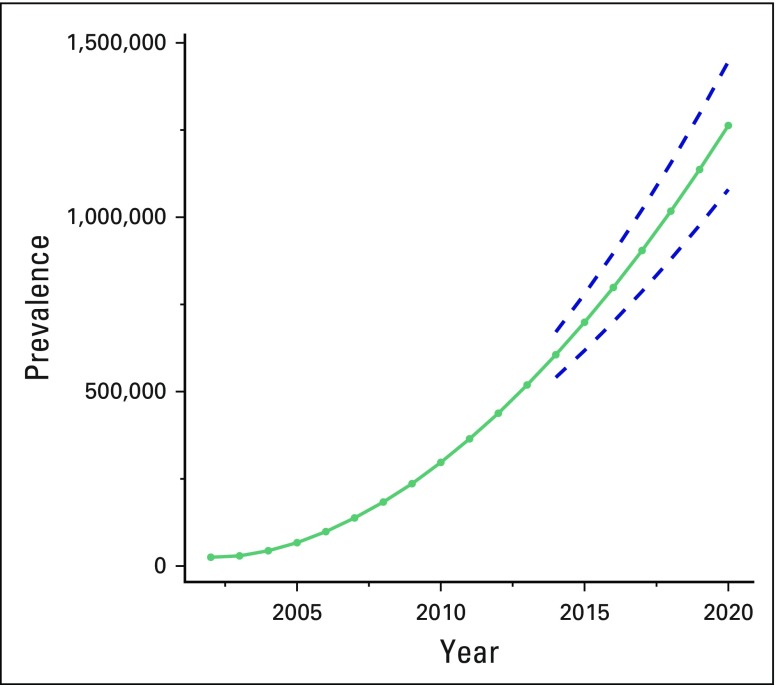
Prevalence estimates of cancer in Mexico for 2020. Data used with permission.^12-14^

With the available data, the estimated cancer mortality rate in Mexico by 2020 will increase to 79 per 100,000 inhabitants (95% CI, 76.51 to 81.48). At the same time, it is estimated that people living with cancer in Mexico will increase annually by 109,500, from 518,891 (95% CI, 463,212 to 574,750) in 2013 to 605,758 (95% CI, 540,665 to 670,852) in 2014, 698,908 (95% CI, 617,851 to 779,965) in 2015, 798,489 (95% CI, 700,067 to 896,912) in 2016, 904,581 (95% CI, 787,323 to 1,021,839) in 2017, 1,017,277 (95% CI, 879,630 to 1,154,924) in 2018, 1,136,672 (95% CI, 976,994 to 1,296,351) in 2019, and approximately 1,262,861 (95% CI, 1,079,419 to 1,446,303) in 2020 (Fig 4).

## DISCUSSION

According to the Mexican National Institute of Statistics and Geography, Mexico in 2012 was a country with a population of greater than 117 million people, 51.2% of whom were women. The mean age of the population is projected to increase from 29 years in 2010 to 31 years in 2020 and to 38 years in 2050. A notable increase in the 15-to 64-year-old age group is estimated, which means that the population most at risk for chronic diseases will be economically active. It is estimated that, by 2030, the Mexican population will begin the final stage of demographic transition, in which sharp declines in mortality, fertility, and growth rates are expected; the culmination of this transition is reduced growth and an aging profile, which will result in public health consequences.

Although limited, the results in this article show the extent of cancer in Mexico. We know that cancer currently is the third leading cause of death in Mexican population; 45.3% of deaths from this group of diseases occur in the economically active population. Approximately half of all cancer deaths were a result of lung, gastric, liver, prostate, breast, or cervical tumors. Incidence data are nearly nonexistent and differ from reported international estimates. However, information about risk factors, including the expected aging of the population, points to possibly substantial increase in the occurrence of new cancer diagnoses in the coming decades.

Mexico is a country with a high prevalence of risk factors associated with the onset of cancer, such as smoking and alcohol use. According to data from the 2011 National Survey of Addictions, the prevalence of active consumption of tobacco cigarettes was 21.7% in the 12- to 65-year old age group and was 12.3% in teenagers. The prevalence of alcoholism in the population ages 12 to 64 years is as high as 51.4%.^20^

Moreover, excess weight and obesity are serious problems in Mexico. In 2012, the combined prevalence was higher among women (73%) than among men (69.4%). For the population younger than age 5 years, the prevalence was 9.7%; for children ages 5 to 11 years, it was 34.4%; and for adolescents (ages 12 to 19 years), it was 33.2%.^21^

The disease burden that cancer will cause seems overwhelming. Currently, the proportion of people age 65 years or older in Mexico is 6%—less than data in published reports for Anglo-Saxon countries in 2011 (ie, 13% in the United States, 16.5% in the United Kingdom, 15.9% in Canada, and 20% in Germany).^22^ The slow demographic transition in Mexico increases the proportion of young working-age people likely to be affected by catastrophic illnesses, such as cancer.^23,24^ However, it is expected that, at the end of the demographic transition, the proportion of older adults will be even greater.

In light of this scenario, the Mexican Health System has developed a series of public policies to reduce potentially modifiable risk factors. A specific antitobacco program^25,26^ has been active since 1986. In 2008, the General Law on Tobacco Control went into effect, and the Nonsmokers Health Protection Law was enacted by the Federal District; this represented the first Mexican law banning smoking in public places.^27-29^

With respect to known infectious risk factors, hepatitis B vaccination was incorporated into the Universal Immunization Program of Mexico in 1999. In addition, since 2009, the Mexican immunization program includes vaccination against human papillomavirus.^30-32^

A number of initiatives to reduce obesity have been launched, and they have resulted in the new National Strategy for the Prevention and Control of Overweight, Obesity, and Diabetes.^33-35^ Despite knowledge about the high prevalence of factors associated with the onset of cancers,^36^ the magnitude of the risk for the Mexican population to develop cancer is still unknown. This is a window of opportunity for cancer research in our country, which will define the potential growth of the epidemic in the medium term in different contexts, and it will define the assessment of implemented primary prevention strategies for its control.

The proportion of all-cause mortality represented by cancer (13%) is still low in Mexico compared with developed nations like the United States (23%), the United Kingdom (29%), and Germany (28%) or with other Latin American countries, such as Argentina (28%) and Brazil (17%). The *Cancer in the Americas: Country Profiles 2013*^37^ report by the Pan American Health Organization shows that the highest rates of cancer mortality in Latin America and the Caribbean are found in Trinidad and Tobago, Cuba, and Argentina, and the lowest mortality rates are in Mexico, Nicaragua, and El Salvador.

In the recent CSA cancer report, Mexico, compared with the other 19 countries of CSA and the United States, ranks 11th of 21 countries for average rates of 30 cancers. Only liver and pancreatic cancers occupy high places (fourth and sixth places, respectively) in the ranking of mortality rates; for breast cancer, Mexico ranks eighth in the region, but the incidence is high and the mortality is low.^11^

In Mexico, the poor impact of actions and interventions for prostate and breast cancers, compared with the actions for cervical cancer, has resulted in the highest mortality for men and women, respectively, from these tumors.^38^ This is similar to what has been reported in other Latin American countries.^39,40^ This differs from data from Canada and the United States, where lung cancer is the leading cause of cancer deaths in both men and women.^41^

Our mortality data by state, with mortality rates between 40 and 80 per 100,000 inhabitants, show heterogeneity in the epidemiologic transition occurring in the country, and the data reveal aberrations in the registry of deaths as a result of cancer—the same inequality in cancer care seen in other Latin American countries—including the effect of a fragmented health system that lacks national public policies for cancer prevention and the absence of an appropriate reference system to ensure early diagnosis and timely treatment of all patients with cancer.

The main limitation of our study is the lack of national epidemiologic information, which hinders the collection of comprehensive data on cancer incidence and mortality. Notwithstanding this huge deficiency, our results are similar to those projected by GLOBOCAN.^13^ Estimates of new cancer cases reported by GLOBOCAN show a 30% increase between 2012 and 2020, whereas we estimated an approximate increase by 43% for the same period.

In addition, the GLOBOCAN system estimated a 32% increase in mortality in the period of 2012 to 2020, compared with a 15.2% increase derived from our calculations. It must be mentioned that our mortality figures for 2020 were estimated on the basis of the number of deaths and the national projections for population growth, whereas GLOBOCAN uses the most recent mortality rates and the population corresponding to the year of a given analysis. This may be the reason for the slight discrepancies found. Our estimations also agree with those reported by Sierra et al^19^ for mortality rates from 2006 to 2010, with only slight differences, although the data from Sierra et al^19^ were based on the World Health Organization mortality database.

Weir et al^42^ estimated that, in the United States, the numbers of new cancer occurrences in women will increase 20.6% between 2010 and 2020; for the same period, new occurrences in men will increase 24.1%. The estimated number of deaths is expected to increase 8.1% in women and 15.2% in men. It should be noted that the estimates of new occurrences are lower than ours and those mentioned by GLOBOCAN. However, the estimates were made for a developed country that has a more robust epidemiologic surveillance system; given that difference, the projection of deaths is similar to our findings.

However, we believe that estimates of cancer prevalence obtained in this study are useful to show the impact that cancer will have in the upcoming years and to assess the medium-term impact of the strategies implemented for the prevention and control of cancer in Mexico.

There is a fundamental need to strengthen the information systems and cancer registries to identify the target population of any developed public policy. Without reliable data sources, it will be impossible to plan preventive measures at all levels and to assess the achieved results. This strengthening should also consider incorporating modestly private sector activities. This will allow us to construct a more complete picture of cancer epidemiology in Mexico.

This analysis is based on available data sources, and it has the potential to alert the Mexican health system about the primary needs for cancer control. This control necessarily includes the creation and establishment of good-quality population-based registries designed to monitor the impeding cancer epidemic and to provide the basis for implementation of actions to limit the increasing burden of cancer in the country.

In conclusion, available data for cancer are incomplete. To develop and implement population-based cancer registries is essential. The authors estimate that the prevalents cases of cancer will be more than one million for the next decade. By knowing the future outlook of cancer in Mexico, health systems will be aware of the challenge than awaits them and can prepare to face them.
